# Biological Activities of Citrus-Derived Extracellular Vesicles on Human Cells: The Role of Preservation

**DOI:** 10.3390/cimb46060347

**Published:** 2024-06-11

**Authors:** Theodora Karamanidou, Konstantinos Krommydas, Maria Karanikou, Dimitrios Tsamos, Konstantinos Michalakis, Dimitris Kletsas, Alexander Tsouknidas, Harris Pratsinis

**Affiliations:** 1 Laboratory for Biomaterials and Computational Mechanics, Department of Mechanical Engineering, University of Western Macedonia, 50100 Kozani, Greecedmech00075@uowm.gr (K.K.); d.tsamos@uowm.gr (D.T.); 2 Laboratory of Cell Proliferation and Ageing, Institute of Biosciences and Applications, National Centre for Scientific Research “Demokritos”, 15341 Athens, Greece; dkletsas@bio.demokritos.gr; 3 Department of Restorative Sciences & Biomaterials, Henry M. Goldman School of Dental Medicine, Boston University, Boston, MA 02118, USA; kmich@bu.edu

**Keywords:** tangential flow filtration, fresh EVs, freeze-thawed EVs, freeze-dried EVs, cell proliferation, Akt phosphorylation, antioxidant activity

## Abstract

Extracellular vesicles (EVs) have been identified as important mediators for cell-to-cell communication. Citrus-based EVs in particular offer an excellent platform for nutraceutical delivery systems, as their endemic cargo includes micronutrients (e.g., ascorbic acid), which contribute to their antioxidant capacity. Despite being extensively investigated as to their therapeutic and diagnostic potential, their cargo is inherently unstable and thus directly affected by their storage and preservation. In this study, EVs were isolated from citrus fruit using tangential flow filtration and evaluated for their physicochemical characteristics, antioxidant activity and effects on human cells. To assess how their isolation and preservation methods affect these properties, the EVs were tested immediately after isolation (from fresh and freeze-thawed juices) or following freeze-drying. A measurable biological effect of cryoprotection on citrus-derived EVs was evident, whether during or after isolation. This was more pronounced in the cell-based assays, ranging from −4% to +32% in human skin fibroblast proliferation. Nevertheless, the effects on human cancer cells varied depending on the cell line. Although these results should be considered preliminary observations, subject to further investigation, it is safe to state that any type of preservation is expected to impact the EVs’ biological activity.

## 1. Introduction

Extracellular vesicles (EVs) released by eukaryotic cells, both metazoal and plantal, have been identified as crucial mediators of cell-to-cell communication in health, disease and other essential biological functions, such as organ homeostasis. These biomolecules are often referred to as exosomes, which are essentially an important EV sub-category of endosomal origin. Exosomes have recently risen to prominence and have been extensively investigated as therapeutical and diagnostical elements due to their diverse nature and their essential contribution in various biological processes, such as inflammation, aging and immunomodulation. The therapeutic and diagnostic potential of EVs is regulated by their complex cargo, including proteins, amino acids, nucleic acids and metabolites [1], which are bound by a lipid bilayer structure, also facilitating the EVs’ safe migration through extracellular spaces [2,3]. This renders EVs as highly sophisticated nanocarriers, whose targeting capacity and uptake can be tuned further through chemical modification techniques, increasing their compatibility to target cells through affinity-based endocytosis [4].

EVs isolated from edible plants (or fruits) offer an excellent platform for nutraceutical delivery systems, not only based on their capacity for cellular communication but also due to functional micronutrients (e.g., ascorbic acid), which are endemic to their structure [5]. Furthermore, the fact that EVs can be isolated from edible plant species via standardized isolation and purification techniques holds a significant advantage over other nanocarriers, as EVs can be used as nutraceutical delivery systems without prior certification (as long as they are not chemically modified). An application of this can be sought in the mediation of oxidative stress through citrus-derived EVs, containing antioxidant components such as ascorbic acid [6].

For all the aforementioned reasons, EVs can become a significant therapeutic tool against various diseases, such as oxidative stress-related conditions, and be an effective mediator with promising antioxidant capabilities.

Oxidative stress is associated with an imbalance between reactive oxygen species (ROS) production and the ability of biological systems to mitigate these chemicals to avoid accumulation, ultimately leading to cellular damage [7]. Numerous studies have stipulated that the anti-inflammatory properties and therapeutic potential of EVs can be attributed to their antioxidant effects, while preserving their cytoprotective properties even at concentrations of up to 50 μM [6]. An extensive review of the antioxidant effects of exosomes and their role in various disorders (e.g., neural-, circulatory-, musculoskeletal-, gastrointestinal-diseases, etc.) attributed the inhibition of excessive ROS production to immunomodulating mechanisms [2].

However, despite the attractiveness of EVs as a new generation of nutraceuticals, they remain subject to limitations associated with their storage and preservation [8,9]. As biomolecule-decorated entities, their nucleic and proteomic cargo is inherently unstable when exposed to ambient or fluctuating temperatures, non-physiological pH values or contaminating nucleases [10].

The hypothesis of this study is that the physicochemical and cytoprotective properties of citrus-based extracellular vesicles are affected by both their isolation/purification and preservation methods; thus, any potential for clinical application would be subject to careful consideration of these parameters. To put this hypothesis to the test, exosomes were isolated from citrus fruit (derived from fresh and freeze-thawed juices) using tangential flow filtration (TFF). The EVs were then evaluated as isolated or freeze-dried with respect to their physicochemical characteristics. Additionally, their bioactive properties were evaluated in terms of antioxidant activity as well as their effect on human cell viability and proliferation.

## 2. Materials and Methods

### 2.1. EV Isolation

Before juice isolation, from citrus sinensis (Newhall Sweet oranges), the fruits were washed meticulously with tap water. For the experimental procedure, 4 middle-sized oranges were cut in half and the juice was extracted (400 mL). Half of the juice extracted (i.e., 200 mL) was stored at −15 °C until further use. The juice was purified using a commercial sieve to remove large impurities. The EV isolation process was based on an innovative TFF technique. In brief, the remaining 200 mL were centrifuged step-wise at 200× *g* (10 min), 2000× *g* (10 min) and 10,000× *g* (30 min) using a BRC-5180UT refrigerated centrifuge from InoviaLabs (Istanbul, Turkey). After the last centrifugation, the supernatant was collected and filtered, firstly by a 1.2 nm fiberglass filter and secondly by a 0.45 nm syringe filter. The sample was additionally filtered and concentrated using TFF (TFF-MV and TFF-Easy, from HansaBioMed Life Science Ltd., Liivalaia, Estonia) to remove impurities, such as agglomerates of proteins, micro-fragments and other organic aggregates.

### 2.2. Physicochemical Characterization

Dynamic light scattering (DLS) was performed with a VASCO 3 DLS analyzer from Cordouan Technologies (Pessac, France), providing information on the particle size distribution profiles. The morphological characteristics of the isolated EVs were assessed using scanning electron microscopy (SEM), performed on a JSM-6610LV device from JEOL (Tokyo, Japan). The EV samples were affixed to carbon conductive tape. A thin layer of gold (10 nm) was applied to each sample using the Quorum Q150R S (Sussex, UK) prior to the SEM examination.

Additionally, UV–vis spectroscopy, implemented via a Cary 60 from Agilent Technologies (Santa Cara, CA, USA), was used to measure the amount of discrete wavelengths transmitted through the EV dispersion. Attenuated total reflectance (ATR), performed on a Cary 630 FTIR Spectrometer from Agilent Technologies (Santa Cara, CA, USA) with a Diamond ATR sampling accessory, was used to analyze the citrus extracellular vesicles.

### 2.3. Antioxidant Potential

To determine the antioxidant potential of orange-derived EVs, a DPPH assay was deployed. The DPPH radical was mixed in methanol, giving a final concentration of 0.05 mM. The evaluation protocol was based on a technique described by Noipa et al. [11]. In brief, three methanol solutions were prepared.

• Solution A, DPPH solution diluted up to 0.01 mM.
• Solution B, EV extract diluted in methanol, to produce different EV concentrations (e.g., 1.13 mg/mL, 2.26 mg/mL, 3.11 mg/mL and 4.1 mg/mL).
• Solution C, EV extract and DPPH solution (0.05 mM) diluted in methanol, producing again the same EV concentrations as Solution B.

All solutions’ absorption was measured at 517 nm using UV–vis spectroscopy. Solution C was measured immediately after preparation, followed by four further measurements after 15, 30, 45 and 60 min, resulting in the samples’ percentile antioxidant activity over time.

### 2.4. Cell Expansion and Maintenance

Human neonatal foreskin fibroblasts AG01523 were obtained from the Coriell Institute for Medical Research (Camden, NJ, USA). Two human cancer cell lines from tumors of different histopathologic origins, i.e., fibrosarcoma HT-1080 and mammary adenocarcinoma MCF-7, were obtained from American Type Culture Collection (ATCC; Rockville, MD, USA). The cells were routinely grown in Dulbecco’s Modified Eagle Medium (DMEM) containing 4.5 g/L (5.5 mM) glucose, 100 IU/mL penicillin, 100 μg/mL streptomycin, and 2 mM glutamine supplemented with fetal bovine serum (FBS; 15% *v*/*v* and 10% *v*/*v* for normal and cancer cells, respectively). Culture media and sera were obtained from Life Technologies Europe BV (Thessaloniki, Greece). The cultures were maintained at 37 °C in a humidified atmosphere (95%) of 5% CO_2_.

The cells were subcultured using a trypsin (0.25%; Life Technologies Europe BV)-citrate (0.3%; Sigma, St. Louis, MO, USA) solution at a 1:2 split ratio [12]. The cell number was assessed by suspending the detached cells in IsoFlow sheath fluid (Beckman Coulter Life Sciences, Indianapolis, IN, USA) and counting them by means of an automated Beckman Coulter Z1 counter. The cells were periodically tested using the MycoProbe Mycoplasma Detection Kit (R&D Systems, Abingdon, UK) and found to be free of mycoplasma.

### 2.5. Cell Viability and Proliferation

The MTT (3-(4, 5-dimethylthiazolyl-2)-2, 5-diphenyltetrazolium bromide) technique, a colorimetric method, was employed to measure cell viability and proliferation. The method is based on the ability of mitochondrial dehydrogenases in living cells to convert soluble yellow tetrazolium salt into insoluble purple formazan crystals [13]. To this end, AG01523 cells were seeded into flat-bottomed 96-well plates at a density of approximately 7000 cells per well in DMEM 15% FBS and left overnight to adhere. The cancer cells were seeded at a density of 5000 cells per well in DMEM 10% FBS. Following cell attachment, the tested EVs’ solutions (at 9.33, 3.11 and 1.04 mg/mL) diluted in the culture medium were added to the cells at the intended concentration for 72 h. At the end of the incubation period, the medium was replaced with MTT (Sigma) dissolved at a final concentration of 1 mg/mL in serum-free, phenol-red-free DMEM for a further 4 h incubation. The MTT formazan was solubilized in 2-propanol (Sigma), and absorbance at a wavelength of 550 nm was measured using a SPARK (Tecan Group Ltd., Männedorf, Switzerland) microplate analyzer [14]. Counting at a reference wavelength of 690 nm was used to correct for plate absorbance. Doxorubicin hydrochloride (Sigma) at the indicated concentration range was used as a positive control.

### 2.6. Cellular Glutathione Levels

Cellular glutathione levels were assessed based on the fluorescent probe monochlorobimane (mCB) [15]. To this end, AG01523 human skin fibroblasts were grown to confluency inside clear-bottomed black 96-well microplates. Then, the tested EVs’ solutions diluted in phenol-red free, serum-free DMEM, as well as the corresponding controls (see below), were added to the cells at the intended concentration and left for overnight incubation. The media were then replaced with Hanks’ Balanced Salt Solution (HBSS; Life Technologies Europe BV) containing 5 μM mCB (MedChemExpress, Monmouth Junction, NJ, USA), the cell cultures were incubated at 37 °C in the dark for a further 4 h, and fluorescence was recorded in a Spark (Tecan) plate reader using an excitation wavelength of 380 nm and emission wavelength of 480 nm. N-acetycysteine (NAC) at 5 mM was used as a positive control [16], while plain growth medium was used as a negative control.

### 2.7. Western Blotting

The tested EVs’ solutions diluted in culture medium supplemented with 0.1% FBS (final concentration 3.11 mg/mL) were added to confluent AG01523 human skin fibroblast cultures for 30 min. Then, the cells were quickly rinsed in cold Tris-buffered saline (TBS) and lysed with the help of 2x-sample buffer containing protease and phosphatase inhibitors (Sigma), as described previously [17]. The cell lysates were scraped into Eppendorf-type tubes, heated for 3 min at 100 °C, sonicated, centrifuged, and the supernatant was collected and stored at −80 °C until use. Cell lysate proteins were separated using SDS-PAGE (10% Bis-Tris polyacrylamide gels) and transferred to polyvinylidene fluoride (PVDF) membranes (Thermo Scientific, Rockford, IL, USA). The membranes were blocked in 5% *w*/*v* nonfat milk in TBS-T (TBS supplemented with 0.05% Tween-20 buffer) for 1 h and incubated overnight with the primary antibody. Subsequently, the membranes were washed three times with 5% *w*/*v* nonfat milk in TBS-T before probing with the appropriate species-specific horseradish peroxidase-conjugated secondary antibody (Sigma). Finally, after washing twice with 5% *w*/*v* nonfat milk in TBS-T and once with TBS-T alone, the immunoreactive bands were visualized by chemiluminescence using a horseradish peroxidase substrate (Immobilon Crescendo Western HRP substrate, Merck Millipore, Darmstadt, Germany) and captured using an LAS-4000 luminescent image analyzer (Fujifilm Manufacturing USA Inc., Greenwood, SC, USA). The primary antibodies used in the study were recognized as phospho-Akt (Ser473), phospho-SAPK/JNK (Thr183/Tyr185) and phosphor-ERK1/2 (Thr202/Tyr204), the first two being purchased from Cell Signaling Technology (Beverly, MA, USA) and the third from BD Transduction Laboratories (Bedford, MA, USA). To validate equal loading, probing with a mouse monoclonal anti-pan-actin antibody (NeoMarkers, Lab Vision, Fremont, CA, USA) was performed. Densitometric analysis was implemented using ImageJ 1.52a [18].

### 2.8. Statistical Analysis

The results are presented as the mean ± standard error of the mean (SEM) of at least two independent experiments. Student’s *t*-test was used for the evaluation of statistically significant differences. Statistical analyses were performed using Microsoft Office Excel Version 2405 (Build 17628.20110) or the online GraphPad *t*-test calculator (https://www.graphpad.com/quickcalcs/ttest1.cfm, 19 April 2024).

## 3. Results

### 3.1. Physicochemical Characterization

**Figure 1:**
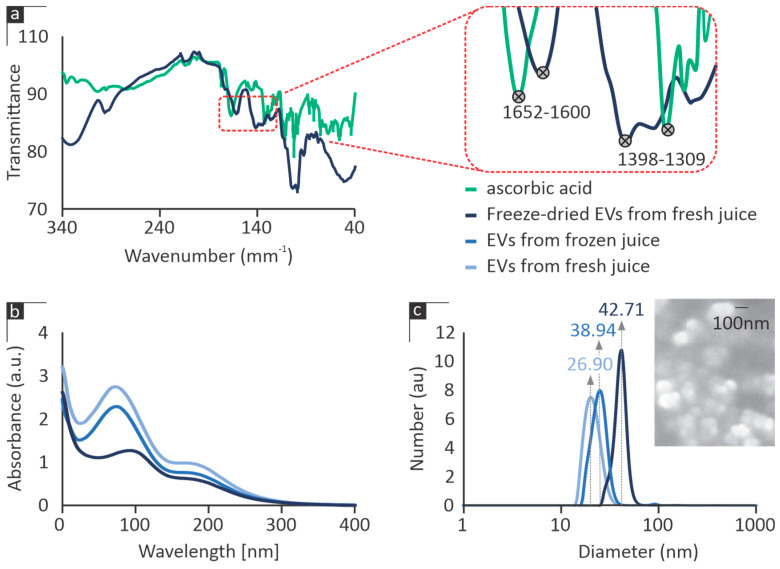
Physicochemical characterization of citrus EVs produced at various isolation/preservation stages. (**a**) Attenuated total reflection spectra of EVs and comparison of their characteristic bands to known functional groups, (**b**) UV-Vis spectra, and (**c**) size distribution as determined using DLS measurements and validated via SEM (showing spherical characteristics).

The stretching and bending frequencies of the molecular groups within the examined EV species were characterized using FT-IR analyses. The documented spectra were then compared to characteristic bands of known functional groups, such as ascorbic acid, as shown indicatively in Figure 1a. Characteristic transmittance peaks at 1600 cm^−1^ and 1398 cm^−1^ demonstrated bending vibrations typical of citric acid bonds with similar absorption peaks at 1652 cm^−1^ and 1309 cm^−1^, respectively. This is indicative of the presence of this well-established antioxidant in all of the examined samples. Other functional groups may also be present in the tested EVs, although their identification is beyond the scope of the manuscript.

UV–vis spectroscopy of the samples revealed similar absorption peaks at wavelengths between 265 and 280 nm, as illustrated in Figure 1b. The intensity of absorbance was clearly affected by the preparation route of the samples, with freshly derived EVs exhibiting a notably higher shoulder peak than EVs extracted from frozen juice, followed by freeze-dried samples.

The size distribution of the isolated EVs, as determined through DLS, is illustrated in Figure 1c. The data indicate the presence of a single monodispersed population within each of the samples. Measurements were repeated in triplicate, always indicating almost identical results. The average size of EVs derived from fresh citrus juice was documented at 26.9 ± 0.11 nm, while freezing of the juice during the isolation process and lyophilization of the final EVs led to vesicles with an increasing particle size averaging 38.94 ± 0.12 nm and 42.71 ± 0.14 nm, respectively. This indicates that both isolation technique and storage influence the physicochemical characteristics of the isolated EVs.

The size of the EVs was validated through SEM imaging, which showed EVs sized similarly to those from the DLS measurements. The spherical morphology of the freeze-dried EVs is indicatively presented in Figure 1c.

### 3.2. Antioxidant Activity

**Figure 2:**
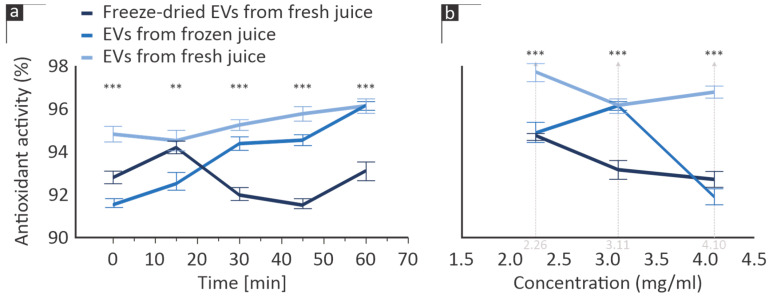
Antioxidant activity (% radical scavenging activity) of citrus EVs produced at various production/preservation stages, (**a**) as a function over time (at a 3.11 mg/mL concentration) and (**b**) as a function of concentration, after 60 min. Error bars indicate standard error. ** *p* < 0.01, *** *p* < 0.001.

The antioxidant activity of citrus-derived EVs, as illustrated in Figure 2 (results are plotted as the average of three measurements performed on each sample), seems to be affected by the isolation method and preservation stages employed. Freshly derived EVs seemed to exert consistent antioxidant activity, whereas the effectiveness of EVs extracted from frozen citrus juice seemed to fluctuate over time, as did the effectiveness of freeze-dried ones.

Interestingly, the antioxidant activity of all samples amounted instantly to more than 95% of their highest capacity (Figure 2a). The dose-dependent mediation of the oxidative response exhibited a similar trend in EVs isolated from fresh juice and freeze-dried samples, with all tested concentrations performing with a 92–97% efficacy (Figure 2b). Nevertheless, the antioxidant activity of the EVs was not proportional to their concentration, stipulating that the best-performing concentration might occur at higher or lower concentrations than the ones examined in this study.

### 3.3. Cell Viability and Proliferation

**Figure 3:**
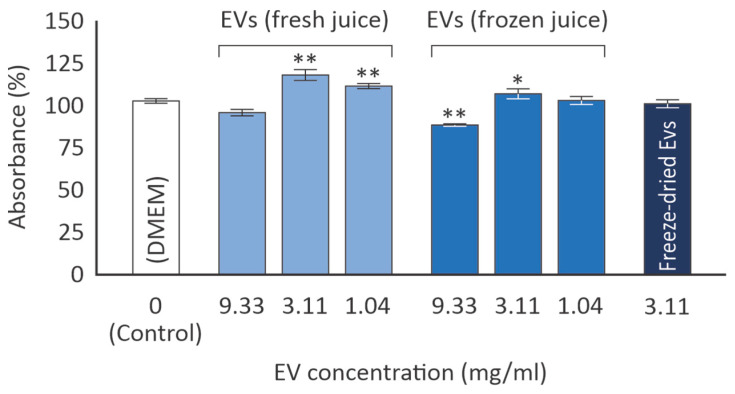
Relative % absorbance of AG01523 cells in DMEM medium (control) vs. incubation with the EVs from fresh and frozen juice at the indicated concentrations for 72 h (mean values from three independent experiments; error bars indicate standard error; * *p* < 0.05, ** *p* < 0.01 based on Student’s *t*-test).

The MTT assay was employed as an indirect method to evaluate cell viability and proliferation. Initially, various concentrations of EVs from both fresh and frozen juice were administered to human skin fibroblasts (AG01523) for 72 h. According to Figure 3, the optimal concentration of both EV groups leading to an increase in viability was 3.11 mg/mL. At this concentration, EVs isolated from fresh citrus juice were more efficient than those from frozen juice (119% ± 3 vs. 107% ± 3; *p* = 0.0179). At a concentration of 1.04 mg/mL, only EVs from fresh juice were active, while the highest concentration of EVs from frozen juice led even to a slight inhibition of viability to 88% of the control (*p* = 0.0001). A concentration of 3.11 mg/mL was chosen for all subsequent experiments in human skin fibroblasts.

Notably, when freeze-dried EVs were tested at 3.11 mg/mL, no statistically significant effect on human skin fibroblast viability was observed (Figure 3).

**Figure 4:**
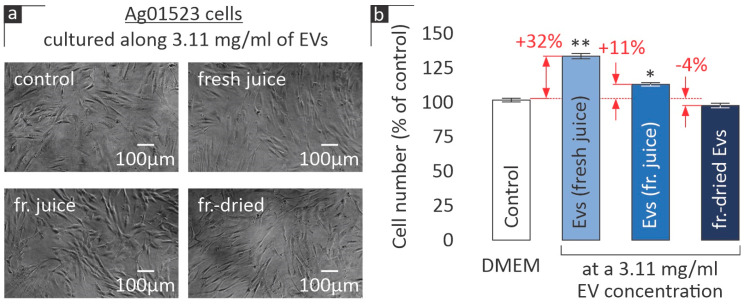
(**a**) Indicative cell morphology, as determined using phase-contrast microscopy after incubation with EVs (3.11 mg/mL) for 72 h. (**b**) Analysis of AG01523 cell proliferation levels when cultured for 72 h along EVs at a 3.11 mg/mL concentration compared to the control medium (mean values from three independent experiments; error bars indicate standard error; * *p* < 0.05, ** *p* < 0.01 based on Student’s *t*-test).

Cell morphology after 72 h of incubation was examined using phase-contrast microscopy, as indicatively depicted in Figure 4a for the control and freeze-dried EV cultures. No significant alterations were observed in either culture.

The trends observed in the MTT experiments were corroborated by the cell count of the AG01523 cultures (Figure 4b). AG01523 cell numbers increased by a respectable 32% ± 2 (*p* < 0.0001) when exposed to EVs (3 mg/mL) derived from fresh citrus juice. Freeze-thawing of the citrus juice prior to the EV isolation also led to cell number increase, but only by 11% ± 2 (*p* = 0.0027) compared to the control. On the other hand, the freeze-dried EVs led to a slight (yet statistically non-significant) inhibition of the cell number.

The effect of EVs on two representative human cancer cell lines (fibrosarcoma HT-1080 and mammary adenocarcinoma MCF-7) notably deviated from the clearly simulating trend observed in Figure 3.

**Figure 5:**
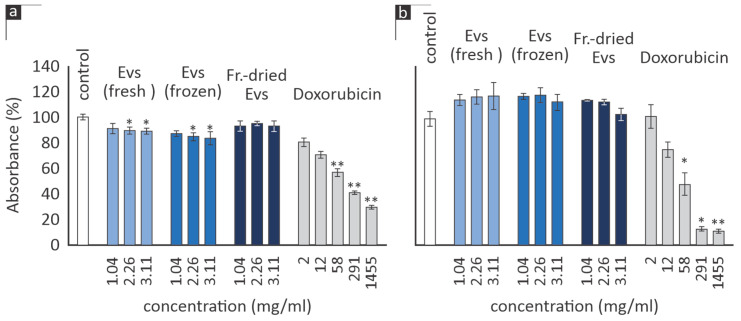
Relative % absorbance of (**a**) HT-1080 and (**b**) MCF-7 cells in DMEM medium (control) vs. incubation with the exosomes groups or the positive control doxorubicin hydrochloride (at the indicated concentrations) for 72 h (mean values from two independent experiments; error bars indicate standard error; * *p* < 0.05, ** *p* < 0.01 based on Student’s *t*-test).

As depicted in Figure 5a, the growth of HT-1080 cells was inhibited by EVs from both fresh and frozen juice at concentrations of 3.11 mg/mL and 2.26 mg/mL, respectively, while the freeze-dried EVs did not significantly affect cell number after incubation for 72 h. On the other hand, EVs did not inhibit MCF-7 cell proliferation, regardless of their origin and the concentration tested (Figure 5b).

### 3.4. Intracellular Signaling Mechanisms

Since citrus-derived EVs possess antioxidant activity (Figure 2), and they enhance human skin fibroblast viability, at least EVs from fresh and frozen juice (Figure 3 and Figure 4), we proceeded to test their effects on the glutathione (GSH) levels in this cell type. GSH is a tripeptide acting in multiple ways to support cellular antioxidant defenses, e.g., through radical scavenging or by acting as an antioxidant enzyme cofactor and by replenishing oxidized antioxidants [19].

**Figure 6:**
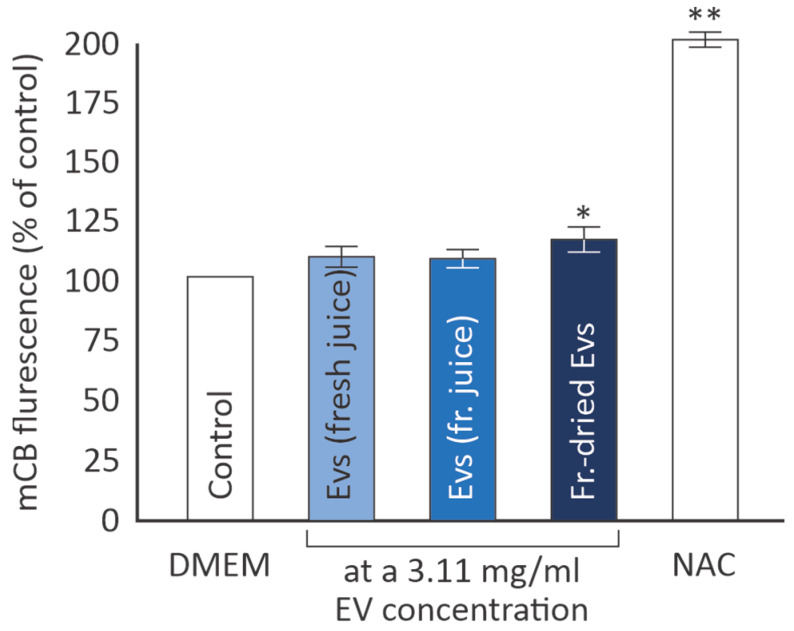
Glutathione levels in AG01523 cell cultures, following an overnight incubation with EVs at a 3.11 mg/mL concentration or with the positive control NAC (5 mM), compared to the control medium (mean values from three independent experiments; error bars indicate standard error; * *p* < 0.05, ** *p* < 0.01 based on Student’s *t*-test).

As shown in Figure 6, EVs from fresh or frozen citrus juice did not statistically significantly affect cellular GSH levels in human skin fibroblasts. On the other hand, the freeze-dried EVs led to a slight, marginally significant GSH increase (116% ± 5, *p* = 0.05), while the positive control N-acetycysteine (NAC) at 5 mM produced a robust response of 200% (±3, *p* < 0.0001), as expected.

Furthermore, since citrus-derived EVs were observed to enhance human skin fibroblast proliferation (Figure 3 and Figure 4), intracellular signaling mechanisms known to be involved in cell proliferation regulation were examined. In particular, the phosphorylation status of members of the phosphatidylinositol 3-kinase/Akt (PI 3-K/Akt) and the mitogen-activated protein kinases (MAPKs) pathways [20,21] was studied following stimulation with the EVs.

**Figure 7:**
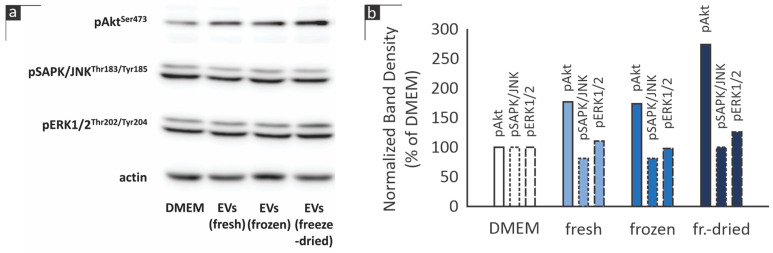
(**a**) Western blot analysis of signaling molecules’ activation in AG01523 cell cultures following a 30 min stimulation with EVs at a 3.11 mg/mL concentration (one representative out of two independent experiments is depicted). In (**b**), the graph depicts densitometric analysis of the Western blot bands following normalization to actin bands’ density and expression as a percentage of the control (DMEM) value.

As shown in Figure 7, all three EV samples tested (from fresh, frozen and freeze-dried citrus juice) induced the phosphorylation of Akt at Ser473 in human skin fibroblasts, while they did not significantly affect SAPK/JNK and ERK phosphorylation.

## 4. Discussion

In recent years, numerous studies have delved into the exploration of plant-derived exosomes, recognizing them as significant therapeutic modalities for addressing various pathological conditions [5]. Citrus-derived extracellular vesicles (EVs) have emerged as noteworthy players in this context, demonstrating their capacity to mitigate oxidative stress in human cells through signaling mechanisms and the delivery of essential micronutrients, such as ascorbic acid [6]. This antioxidative effect has been linked to specific lipids and membrane proteins, whose nanoscale properties may confer functional benefits [22].

Furthermore, investigations have revealed that citrus-derived EVs possess the capability to impede the upregulation of ICAM1 or HMOX-1 in response to stimuli, suggesting a protective role in the activation of inflammatory and oxidative stress pathways [23].

Our observation that citrus-derived EVs stimulate the proliferation of human skin fibroblasts concurs with research advocating for the non-toxic impact of these vesicles on intestinal epithelial cells [23]. This recent study provided evidence that EVs are internalized by these cells within 6 h of incubation, leading to the modulation of gene expression in critical pathways. This includes genes associated with inflammatory responses, such as HMOX-1 and ICAM1, as well as those related to the restoration of intestinal permeability, including claudins and occluding [23]. In their study, Bruno et al. [23] identified two populations of citrus-derived EVs during their DLS measurements, presenting their intensity with respect to their diameter. As scattering intensity is proportional to the 6th power of the particle radius, the larger EV population (despite contributing more to scattering intensity) is likely underrepresented in terms of particle count compared to the population of the smaller-sized EVs. This was corroborated by their TEM measurements [23], indicating only smaller-sized EVs, with dimensions similar to the ones isolated here and presented in Figure 1c. It should be noted that DLS measurements in Figure 1c are presented as a number distribution, i.e., the relative proportion of the number of differently sized particles (instead of how they contribute to intensity). These insights underscore the multifaceted role of citrus-derived EVs in influencing cellular processes, contributing to their potential therapeutic relevance.

This is in good agreement with the results presented here based on both their composition and biological activity. Oxidative stress is of significant importance, as it has been identified as a key component of disorders at the cellular, tissue and organ levels [24,25]. This can be attributed to the fact that the accumulation of free radicals directly affects the metabolic activity of cells, which is vital to the host tissue/organ pathophysiology [26]. Both in vivo and in vitro literature indicates that the antioxidant properties of EVs are considered a major contributor to their anti-inflammatory and cytoprotective potential.

While it is evident that further research is imperative, recent studies have posited that the antioxidant activity of plant-derived EVs may lead to the upregulation of genes such as HO-1, NAO1, GCLM and GCLC (the latter two genes being crucial for GSH biosynthesis), underscoring their significance as promoters of antioxidant mechanisms [22].

Furthermore, owing to their hydrophilic/hydrophobic domains, plant-derived EVs have been suggested to possess inherent protective mechanisms against oxidation in biological systems. This observation accentuates their potential as promising nanocarriers for future applications [22].

The data presented here indicate that citrus-derived exosomes exert a beneficial effect on human skin fibroblast viability (Figure 3) and induce the proliferation of this cell type (Figure 4), suggesting a putative role of these EVs in wound healing and/or cosmetic applications. In a recent study on the stability of mammalian cell-derived EVs, supplementation of the buffer with human albumin and trehalose was shown to significantly stabilize EVs [9]. In our hands, the observed activity of plant cell-derived EVs may be stabilized due to the albumin contained in FBS. Based on our data, we cannot be sure whether the effects of EVs on human skin fibroblasts are related to the EVs’ antioxidant potential or to other mechanisms yet to be determined. In an attempt to identify whether EVs affect the levels of GSH in human skin fibroblast cultures, only freeze-dried EVs were observed to marginally induce GSH levels (Figure 6). On the other hand, all samples from citrus-derived EVs were found to induce the phosphorylation of Akt kinase in human skin fibroblast cultures, 30 min following stimulation (Figure 7). This kinase is implicated in the regulation of cell proliferation and survival [21]; hence its activation may mediate the EV-induced proliferation of human skin fibroblasts (Figure 3 and Figure 4). Further research will be necessary to detect other possible upstream and downstream targets of EVs.

Interestingly, citrus-derived essential oils have been shown to exert an antiproliferative effect on a panel of malignant and normal cell strains, among the latter being human primary gingival fibroblasts [27]. This may either indicate that the exosome preparations used in the current study do not contain the above-mentioned antiproliferative essential oils or simply reflect the different proliferative responses of skin vs. gingival fibroblasts [28]. The latter seems more probable in light of the fact that citrus limon-derived nanovesicles have been found to inhibit cancer cell proliferation [29]. Moreover, the EVs tested in the present study were also found to inhibit the HT-1080 fibrosarcoma cell line, while not affecting the MCF-7 mammary adenocarcinoma cells (Figure 5), reinforcing the idea that different cell types may respond in a different manner. These results should also be viewed in light of the variability in the antiproliferative activity of juice samples from different *Citrus* subspecies tested on various cell lines, including MCF-7 [30]. While fibroblasts can be considered the normal counterpart of fibrosarcoma cells, the fact that no normal mammary cells have been examined can be considered a limitation of the present study. Interestingly, the antiproliferative effects of grapefruit-derived nanovesicles on the A375 human melanoma cell line have also been correlated to Akt phosphorylation inhibition [31], as opposed to the stimulation of Akt phosphorylation by citrus-derived EVs and its correlation to the induction of human skin fibroblast proliferation observed in the present study.

In recent findings, Kim et al. (2021) determined the dose-dependent effectiveness of plant-derived EVs [32], a trend that was documented in this work as well (Figure 2b). Despite this, the application of EVs towards the mediation of these disorders is halted by their susceptibility to environmental conditions, with several researchers reporting that cryopreservation and lyophilization decreases their bioactivity, stipulating a similar effect on their antioxidant activity [33].

The results presented here are indicative of the fact that the biological effect of EVs is dependent on their preservation method. Freeze-drying after their isolation, or extracting them from previously frozen juice, had a measurable effect on both (A) their antioxidant activity (Figure 2) and metabolic assays (Figure 3) and (B) their influence on cell viability and proliferation (Figure 4). Despite these preliminary observations, it is safe to state that any type of preservation (in the absence of a cryoprotectant or similar intervention) seems to significantly impact the EVs’ biological activity, which may be attributed to the destruction of some of their surface proteins, e.g., due to freeze-thawing. A limitation of our study is the lack of structural observations of EVs in the three examined states. Transmission electron microscopy data could arguably provide additional insight into the effectiveness of preservation techniques and will thus be considered in our future work.

Another noteworthy aspect of this study lies in the use of TFF for the isolation of EVs instead of traditionally employed techniques. According to recent literature, the conventional approaches for the isolation of EVs include several lengthy centrifugation/ultracentrifugation steps [5], thus rendering their commercial application challenging. The reduction of these centrifugation steps to below 1 h, combined with TFF, provides an efficient and flexible alternative to conventional isolation routes, as it represents a rapid method with high yield rates. This was stipulated in a study investigating the isolation of EVs from human adipose-derived stem cells, utilizing differential centrifugation and TFF, demonstrating a threefold increase in the volume of purified EVs for the latter [33]. We therefore chose to focus on the isolation utilizing TFF, a filtration technique widely used for the separation and purification of biomolecules [34] but with great potential for exploitation in other scientific areas as well.

While acknowledging that the findings presented in this study serve as preliminary insights necessitating more in-depth investigations, we anticipate that a comprehensive understanding of the capabilities of citrus-derived EVs should also encompass the characterization of their protein and RNA content.

## Data Availability

The data presented in this study are contained within the article.
